# Quantifying Inter-Laboratory Variability in Stable Isotope Analysis of Ancient Skeletal Remains

**DOI:** 10.1371/journal.pone.0102844

**Published:** 2014-07-25

**Authors:** William J. Pestle, Brooke E. Crowley, Matthew T. Weirauch

**Affiliations:** 1 Department of Anthropology, University of Miami, Coral Gables, Florida, United States of America; 2 Departments of Geology and Anthropology, University of Cincinnati, Cincinnati, Ohio, United States of America; 3 Center for Autoimmune Genomics and Etiology, and Divisions of Biomedical Informatics and Developmental Biology, Cincinnati Children's Hospital Medical Center, Cincinnati, Ohio, United States of America; Museo Nazionale Preistorico Etnografico ‘L. Pigorini’, Italy

## Abstract

Over the past forty years, stable isotope analysis of bone (and tooth) collagen and hydroxyapatite has become a mainstay of archaeological and paleoanthropological reconstructions of paleodiet and paleoenvironment. Despite this method's frequent use across anthropological subdisciplines (and beyond), the present work represents the first attempt at gauging the effects of inter-laboratory variability engendered by differences in a) sample preparation, and b) analysis (instrumentation, working standards, and data calibration). Replicate analyses of a ^14^C-dated ancient human bone by twenty-one archaeological and paleoecological stable isotope laboratories revealed significant inter-laboratory isotopic variation for both collagen and carbonate. For bone collagen, we found a sizeable range of 1.8‰ for δ^13^C_col_ and 1.9‰ for δ^15^N_col_ among laboratories, but an interpretatively insignificant average pairwise difference of 0.2‰ and 0.4‰ for δ^13^C_col_ and δ^15^N_col_ respectively. For bone hydroxyapatite the observed range increased to a troublingly large 3.5‰ for δ^13^C_ap_ and 6.7‰ for δ^18^O_ap_, with average pairwise differences of 0.6‰ for δ^13^C_ap_ and a disquieting 2.0‰ for δ^18^O_ap_. In order to assess the effects of preparation versus analysis on isotopic variability among laboratories, a subset of the samples prepared by the participating laboratories were analyzed a second time on the same instrument. Based on this duplicate analysis, it was determined that roughly half of the isotopic variability among laboratories could be attributed to differences in sample preparation, with the other half resulting from differences in analysis (instrumentation, working standards, and data calibration). These findings have serious implications for choices made in the preparation and extraction of target biomolecules, the comparison of results obtained from different laboratories, and the interpretation of small differences in bone collagen and hydroxyapatite isotope values. To address the issues arising from inter-laboratory comparisons, we devise a novel measure we term the Minimum Meaningful Difference (MMD), and demonstrate its application.

## Introduction

**Figure 1:**
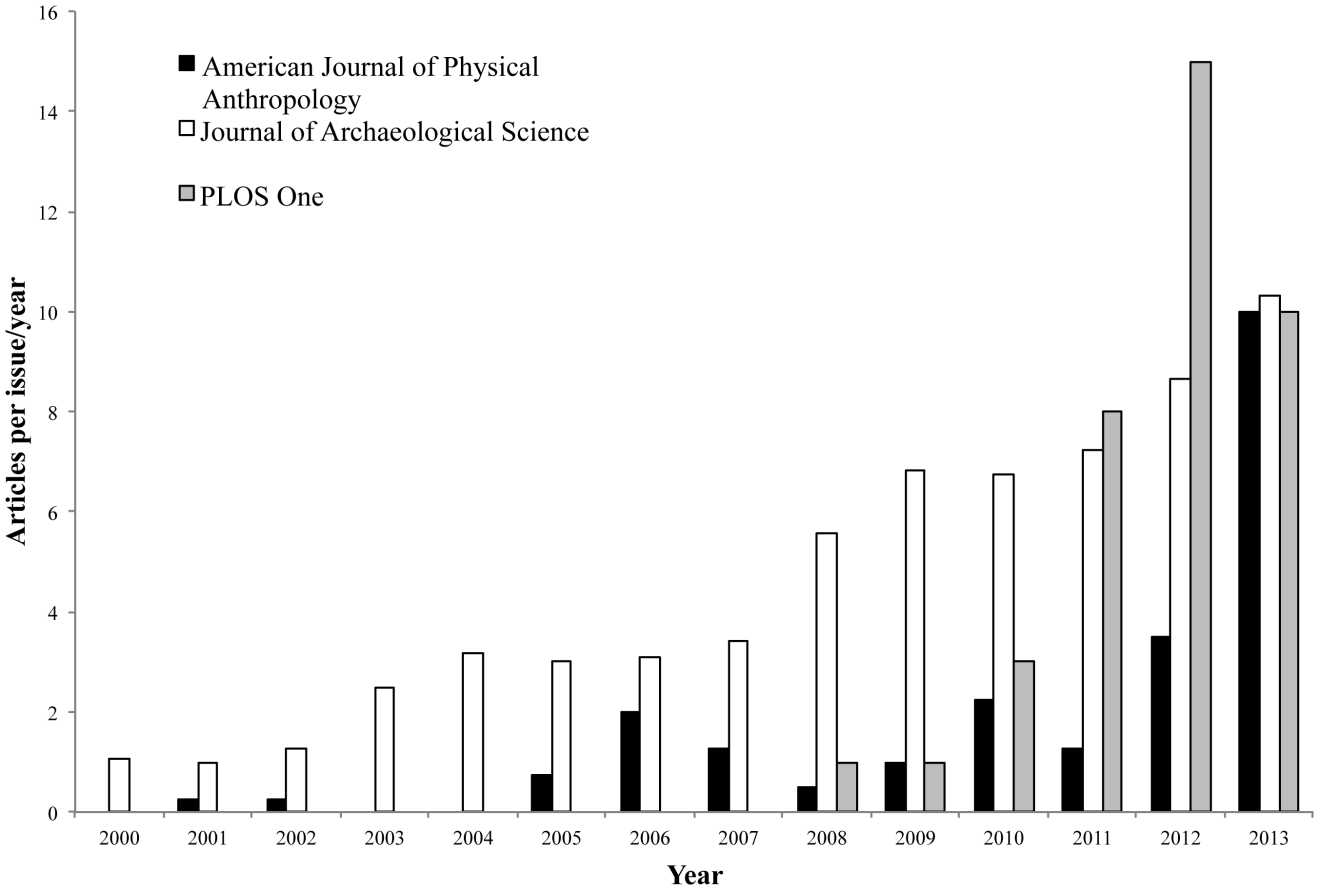
Frequency of publication of articles on archaeological bone stable isotope analysis in American Journal of Physical Anthropology, Journal of Archaeological Science, and PLoS One. Marked increase in frequency is evident.

The past thirty years have witnessed an explosive increase in the ubiquity of stable isotope analysis of osseous remains in the fields of archaeology, paleoanthropology, and paleoecology (Figure 1). Indeed, stable isotope analysis of preserved osseous tissues has become a mainstay of paleodietary and paleoenvironmental reconstruction across anthropological subdisciplines. However, this growth in popularity has outpaced validation of the method's assumptions in at least one key area – the assessment of inter-laboratory variation. The present work aims to rectify this lacuna through experimental establishment of the degree and possible causes of inter-laboratory variation in stable isotope signatures of ancient bone collagen (col) and hydroxyapatite (ap).

The importance of stable isotopes for archaeology was first realized by Robert Hall in the late 1960s when he noted anomalously young radiocarbon dates produced by maize or any other species enriched in ^13^C [1], leading him to posit the utility of stable isotope analysis for the differentiation of archaeological browsers and grazers [2]. The first practical application of stable isotope analysis to the study of ancient human diet did not come until 1977 [3]. In this first study [3], and many thereafter [4]–[7], the main matter of concern was the timing of the introduction of maize agriculture, an event that is fairly obviously evidenced by a dramatic enrichment of consumers' collagen and hydroxyapatite δ^13^C signatures. Shortly after this first publication, DeNiro [8] established the fundamentals of δ^13^C_col_ and δ^15^N_col_ in controlled diet experiments with a variety of animals [9], [10]. He then used these two isotope systems of bone collagen to demonstrate a diachronic dietary shift among the prehistoric inhabitants of the Tehuacan Valley of Mexico. More or less contemporaneously, Tauber [11] used collagen carbon isotope values in his study of prehistoric and historic Danish fishers and farmers, Chisholm and colleagues [12] studied the exploitation of salmon by Northwest Coast Amerindians, Schoeninger and colleagues [13]–[15] demonstrated that both δ^13^C_col_ and δ^15^N_col_ values could be used to discriminate between habitual consumers of marine versus terrestrial foodstuffs, and Ambrose documented the importance of both diet and environment on collagen isotope values [16]–[19].

Paleoanthropological and paleoecological applications of stable isotope analysis have a history of comparable duration, although hydroxyapatite of bone, and more often dental enamel, has been the target osseous biomolecule. In the early 1980s, Sullivan and Krueger [20], [21] outlined the basics of stable isotope paleodietary reconstruction from biological apatites (δ^13^C_ap_), and realized the potential for the technique's application to specimens from “well back into the Pleistocene” [20∶335]. With Lee-Thorp and Van Der Merwe's [22] confirmation that δ^13^C_ap_ values of dental enamel preserved biogenic signatures, the die for such work was cast (although see also [23]). Focusing on carbon isotopes in the inorganic fraction of bone (and more often tooth enamel [24]), various studies have pushed back the temporal horizon of stable isotope analysis well into the Miocene and earlier (see [25]–[29] for review of the pertinent paleoanthropological literature and recent examples).

In the four decades since these first applications, isotope analysis of human (and hominid) dental and skeletal remains has become commonplace. Indeed, Figure 1 demonstrates clearly the method's increasing popularity over the past thirteen years, as represented by the number of publications in three topical journals.

Curiously, however, while the pace of archaeological and anthropological applications of stable isotope analysis has increased, the validation of the technique's assumptions has lagged behind. There have been numerous studies of relevant methodological issues, including isotopic routing [30]–[36], controlled diet experiments [31], [37]–[40], variability among individual laboratory preparation techniques [41]–[44], the causes and consequences of diagenetic and taphonomic change [24], [45]–[58], and the importance of consistent data normalization and calibration procedures for inter-laboratory comparability [59], [60]. However, to the best of our knowledge, there has never been a controlled study assessing the amount of inter-laboratory variation or the degree to which inter-laboratory variation stems from differences in preparation/extraction methods versus difference in analytical instrumentation and data calibration. The present work is intended to remedy these obvious lacunae in our knowledge and assess the confidence in which comparisons of results from different laboratories might be held. This represents a crucial step in assessing just how (dis)similar the conclusions of two laboratories might be when analyzing the same source materials.

The results of this study suggest that, in general, isotopic data from bone collagen (δ^13^C_col_, δ^15^N_col_) derived from different laboratories are directly comparable. However, the direct comparison of isotopic data derived from bone hydroxyapatite (δ^13^C_ap_, δ^18^O_ap_) is more dangerous because variability engendered by differences in pretreatment, analysis, and standardization is of a far greater magnitude. To remedy this issue, we introduce what we have termed the Minimum Meaningful Difference (MMD) value, which serves as an empirically derived threshold by which the significance of values obtained in different laboratories might be judged. In the end, the results of this study have serious implications for choices made in the preparation and extraction of target biomolecules, the comparison of results obtained from different laboratories, and the interpretation of small differences in bone collagen and hydroxyapatite isotope values.

## Methods and Materials

The fundamental premise of the present study is that the best assessment of inter-laboratory variability in stable isotope analysis would require replicate preparation and analysis of the same demonstrably ancient bone sample by a large number of participating laboratories. As such, the initial four major methodological components were: 1) identification and sub-sampling of a suitable ancient human bone sample, 2) verification of this bone's antiquity, 3) recruitment of a representative cohort of participating laboratories, and 4) construction of a rigorous survey and reporting regime by which both laboratory methods and results could be compiled in a manner that would facilitate subsequent statistical analysis. The goal of this work is to characterize the amount of variation present among laboratories rather than comment on “better” or “worse” preparation methods or analytical facilities.

In 2011, one of us (WJP) obtained a presumably ancient unprovenienced human femoral diaphysis from the Museo Gustavo Le Paige in San Pedro de Atacama, Chile. All necessary permits were obtained for the described study (Consejo de Monumentos Nacionales Ord. No. 3682/12, FONDECYT No. 1120376), which complied with all relevant regulations, and the field studies did not involve endangered or protected species. This specimen was judged to be appropriate based on its apparent excellent state of preservation (which is typical of intentionally buried ancient human bone from this hyperarid region of Chile), large size (>100 g), and likely ancient date.

The specimen was AMS ^14^C dated at the University of Arizona NSF-AMS facility following their established protocols for ^14^C dating of bone (acid-base-acid pretreatment, gelatinization, filtration, graphitization). The resulting AMS date for this specimen (laboratory #AA99865) is 1728±47 ^14^C years before present (δ^13^C -17.3‰), This equates to a 2-sigma calibrated age range of 238–470 cal AD when calibrated using Calib 7.0 and the SHCAL13 southern hemisphere terrestrial curve [61], [62].

Subsequent to radiocarbon dating, the authors solicited forty-six archaeological and paleoecological isotope laboratories in order to assess their willingness to participate in this study. Interested laboratories were informed that they would be provided with sufficient sample material to prepare and analyze at least three collagen and three hydroxyapatite replicates (although it was understood that not all laboratories would be able to comply with a full set of both collagen and apatite measurements). While this study was intended to document variation in the analysis of both collagen and hydroxyapatite, participants were asked to perform both types of analysis only if this was routine for their laboratory. In addition to isotopic data (δ^13^C, δ^15^N, δ^18^O), participating laboratories would be expected to provide details on pretreatment and analytical methods, as well as sample preservation assessments (e.g., sample yield, elemental values, amino acid analysis, FTIR spectra, etc.). All potential participants were informed that while their participation in the project would be made known, laboratory attributions of individual results would be kept confidential and all publicly disseminated data would be presented using randomly generated designators. However, in order for each participating laboratory to be able to assess its results compared to those for other study participants, the respective PI's were informed that they would be provided, at the conclusion of the study, with a full complement of the study's results with their data indicated.

Of the forty-six solicited laboratories, twenty-one (46%) ultimately committed to participate (Table 1). When laboratories provided reasons for not participating, they most often cited factors such as cost and time, although other laboratories declined on the basis that they were no longer performing such analyses. Based on the number of participating institutions, we used a handheld Dremel rotary tool equipped with a diamond cutoff wheel to divide the femur into 112 pieces, each weighing approximately 0.75 g. This large number of samples allowed each laboratory to receive five separate individual samples drawn at random from the overall assemblage of 112 pieces, thereby randomizing intra-bone variability and controlling for any random error engendered by differences in sample pretreatment within each laboratory.

**Table 1:** Participating institutions and laboratory PIs.

Institution	Laboratory PI
Arizona State University	Knudson
California State University, Chico	Bartelink
Free University, Amsterdam	Laffoon
Max Planck Institute	Richards
Northern Arizona University	Kellner
Notre Dame University	Schurr
Oxford University	Hedges
University of California, San Diego	Schoeninger
University of California, Santa Cruz	Koch
University of Cincinnati	Crowley
University of Florida	Krigbaum
University of Idaho	Kohn
University of Illinois, Chicago	Pestle
University of Illinois, Urbana	Ambrose/Fort
University of Miami	Pestle
University of Munich	Grupe
University of Rochester	Higgins
University of South Florida	Tykot
University of Tübingen	Bocherens
University of Utah	Cerling
University of Wyoming	Martínez del Rio

In addition to receiving five bone samples, each participating laboratory was provided with an instruction letter requesting that they prepare all five replicate samples using the same standard laboratory method and four standardized survey forms (Figures S1–S4) to use for recording (as appropriate): collagen preparation methods, collagen results, hydroxyapatite preparation methods, and hydroxyapatite results. The use of such standardized forms was intended to maximize comparability of laboratory protocols and to streamline statistical analysis. Some of the participating laboratories did not follow instructions to process all 5 samples or to do so with identical pretreatment.

Summaries of the collagen and hydroxyapatite protocols for each laboratory are provided (using anonymous identifiers) in Tables S1 and S2 (Supporting Information). Twenty of the twenty-one participating laboratories performed collagen extractions, with one laboratory, Laboratory D, performing two different kinds of extractions. Sixteen of the twenty-one laboratories extracted and analyzed hydroxyapatite, with one laboratory, Laboratory N, performing two different kinds of extractions. It should be immediately evident that while there are some broad similarities in sample preparation across laboratories (for example, twenty of the twenty-one collagen preparations (95%) were performed using hydrochloric acid (HCl) as the demineralizing agent), the variation in particle size, reagent concentrations, treatment times, temperature, etc., is substantial. The number and diversity of variables makes identification of particular causes of variability challenging (see discussion below), however we were able to identify protocols that overall yield more (or less) similar results.

To control for at least one potential source of variability, isotopic analysis, we reanalyzed as many samples as possible on one instrument. Eighteen of the twenty laboratories that performed collagen extractions (90%), and eleven of the sixteen laboratories that extracted hydroxyapatite (69%), returned aliquots of prepared material for reanalysis. Three aliquots of collagen and hydroxyapatite (when available) were selected from each laboratory's returned samples for reanalysis. Collagen samples were reanalyzed at the UC-Davis Stable Isotope Facility using a PDZ Europa ANCA-GSL elemental analyzer interfaced to a PDZ Europa 20–20 isotope ratio mass spectrometer (Sercon Ltd., Cheshire, UK). Elemental concentration was standardized by reference to Glutamic Acid, and stable isotope composition was standardized by reference to bovine liver, nylon, and USGS-41 Glutamic Acid. Hydroxyapatite samples were re-analyzed in the Stable Isotope Geochemistry Stable Isotope Laboratory at the Rosenstiel School of Marine and Atmospheric Sciences at the University of Miami using a Kiel-IV Carbonate Device (Thermo-Electron, Bremen, Germany) coupled to a Thermo-Finnigan Delta^Plus^ (Thermo-Electron, Bremen, Germany), and standardized in reference to NBS-19 (TS-Limestone). These duplicate analyses allowed us to independently assess the degree to which isotopic variability resulted from pretreatment versus analysis.

In addition to the use of a battery of well-established statistical analyses (z-score calculation, t-test, ANOVA, Levene's test for equality of variance, Pearson's bivariate correlation, all performed using SPSS v.20 [IBM, New York, USA]), we also used heat maps to visually identify which pretreatment protocols clustered together (i.e., produced similar results). Heat maps visually display data patterns by assigning a gradation of color to numerical values. The heat maps depict the difference in the values obtained by each pair of labs (yellow means no difference, with increasingly red values getting more different). Both axes were clustered using average linkage hierarchical clustering, with Euclidean distance as the distance metric. Heat maps were generated using the Genesis software package developed by Alexander Sturn and Rene Snajder (available freely at http://genome.tugraz.at/genesisclient/genesisclient_description.shtml). Significance for all analyses was set at α = 0.05.

Finally, we developed a novel metric for the evaluation of inter-laboratory variation, the Minimum Meaningful Difference (MMD). The intent of this metric is to establish a means by which to evaluate isotopic results obtained from different laboratories, or when comparing newly obtained results to previously published values in the literature. Our hope is that these values will be treated as an experimentally generated threshold value that one could quickly use when evaluating whether newly generated isotopic data are significantly more enriched or depleted than another laboratory's results or previously published isotopic data. This metric is far more meaningful than a simple t-test, for example, as it explicitly takes into account inter-laboratory variability.

The development of MMDs assumed that the values obtained in the course of the present study are representative of the possible isotope values that might be obtained from any laboratory currently performing such analysis. Minimum Meaningful Differences were calculated by adding the average pairwise inter-laboratory difference for each isotope system plus four times the average of the standard deviations obtained by each laboratory participating in the present study (we used four times the standard deviation for each laboratory in order to account for 95% of the laboratory error from both laboratories in each pairwise comparison). Using this value, a researcher can evaluate with ∼95% confidence the likelihood that a newly obtained isotope value is different from another value as a consequence of *bona fide* biogenic differences rather than laboratory pretreatment and analysis.

## Results and Discussion

### Collagen

Although the present work does not focus on the most-commonly employed indicators of collagen quality (collagen yield, weight %C, weight %N, and atomic C:N), we present these for comparability with other studies. Across all laboratories, the respective values for these metrics were: collagen yield = 16.4±7.9% (the large range of which is explained, at least in part, by the fact that some laboratories employed ultrafiltration whereas the majority did not), weight %C = 41.7±5.3%, weight %N = 15.2±1.9%, and atomic C:N ratio = 3.2±0.1. These data robustly confirm the excellent quality of preservation of the collagen in the selected specimen.

**Figure 2:**
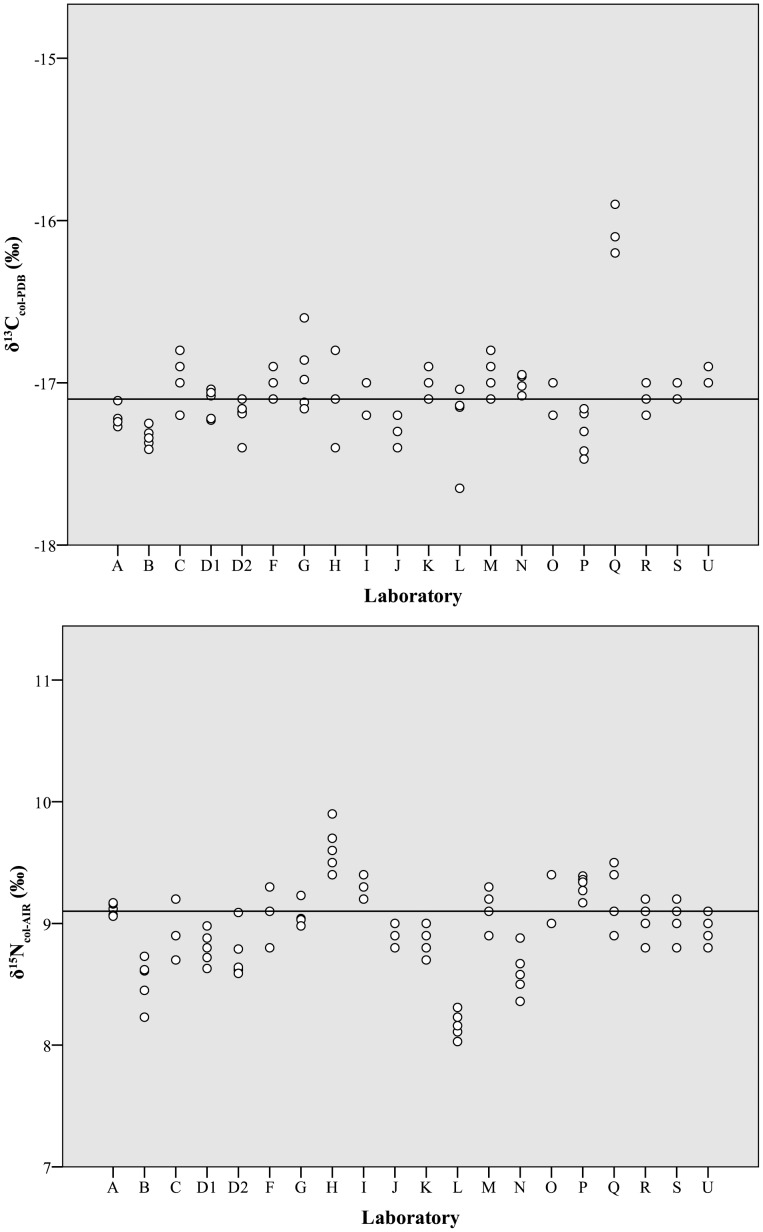
Distribution of initial δ^13^C_col_ (top) and δ^15^N_col_ (bottom) values by laboratory. Dots represent individual analyses and solid horizontal lines represent the median values for all participating laboratories (−17.1‰ for δ^13^C_col_ and 9.1‰ for δ^15^N_col_).

**Figure 3:**
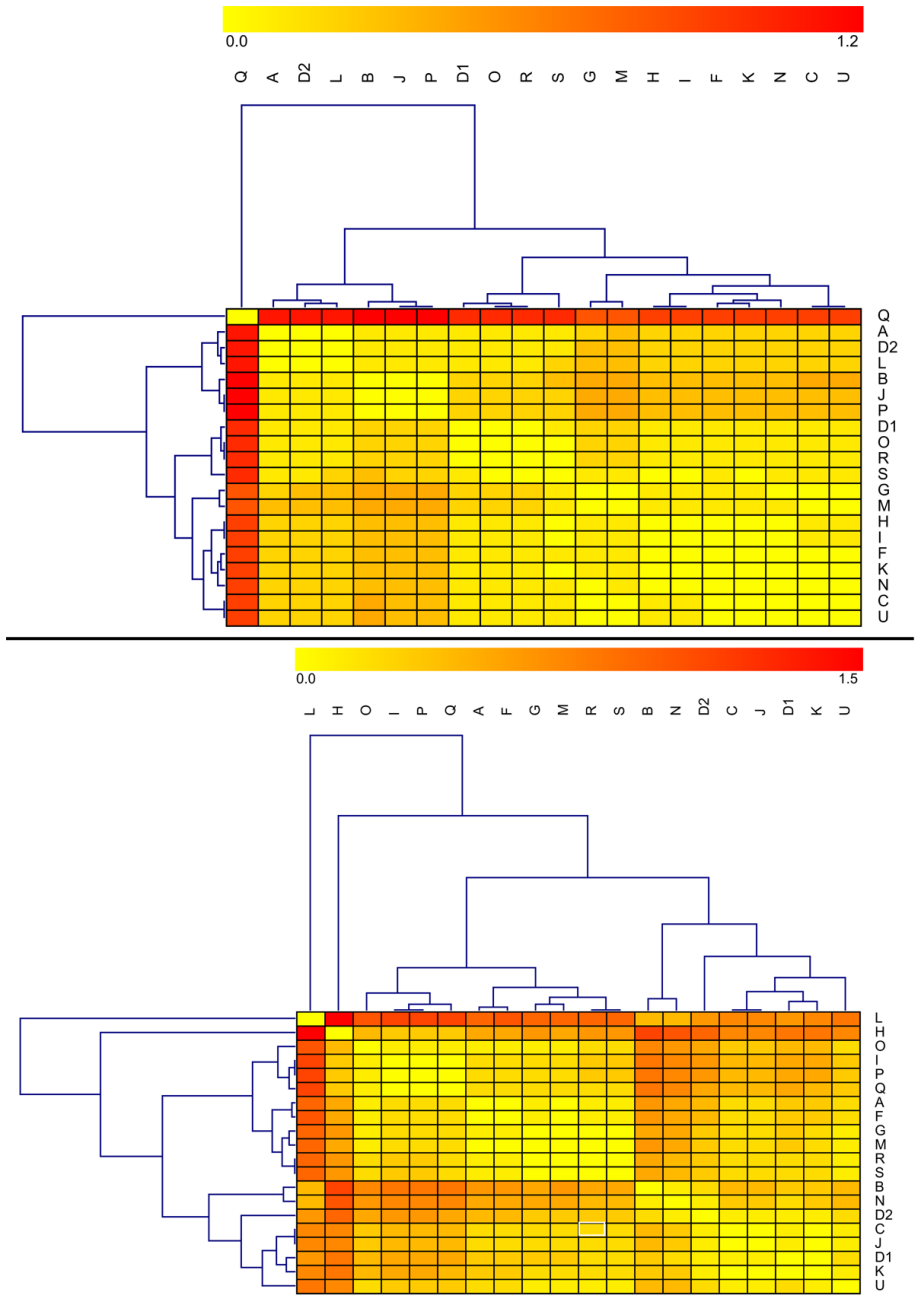
Heat maps of initial δ^13^C_col_ (top) and δ^15^N_col_ (bottom) values by laboratory. Each entry in the matrix depicts the difference in values obtained between the given pair of laboratories. The key is provided at the top – large differences are red, and minimal differences are yellow. Rows and columns have been clustered in order to place similar laboratories near each other (clusters are indicated by the trees above and to the left of the heat maps) – see Methods.

**Figure 4:**
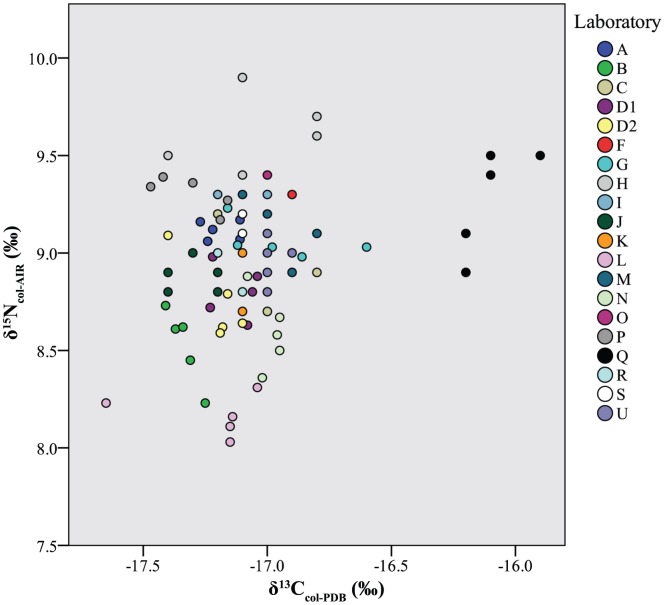
Scatterplot of individual sample δ^13^C_col_ and δ^15^N_col_ values presented for each laboratory. Outlying δ^13^C_co_ values of Laboratory Q are particularly evident.

Across all laboratories, δ^13^C_col_ values averaged −17.0±0.3‰ and had an overall range of 1.8‰ (Table 2, Figure 2, top). Of the ninety-six measured values, six were apparent outliers (note red cells in Figure 3, top): one from Laboratory L (z-score −2.1 [p = 0.02]) and all five from Laboratory Q (z-scores from 3.0 [p<0.01] to 4.1 [p<0.01]). Overall, the laboratories cluster into four distinct groups, with Laboratory Q as a clear outlier (Figure 2, top, Figure 3, top). Nitrogen isotope values averaged 9.0±0.3‰, with an overall range of 1.9‰ (Table 2, Figure 2, bottom). Of the ninety-six measured δ^15^N_col_ values, seven were outliers (with z-scores greater than 2.0 [p = 0.02]): One from Laboratory B, two from Laboratory H, and four from Laboratory L (note red cells in Figure 3, bottom). Four major δ^15^N_col_ groups emerged, with two clear outliers (Laboratories L and H) (Figure 3, bottom). A statistically significant, but overall weak, Pearson correlation (r = 0.26, p = 0.01) was observed between δ^13^C_col_ and δ^15^N_col_ values (Figure 4).

**Table 2:** Results of initial analysis of bone collagen.

Lab	δ^13^C_col-VPDB_ (‰)							δ^15^N_col-AIR_ (‰)						
	R1	R2	R3	R4	R5	Mean	sd	R1	R2	R3	R4	R5	Mean	sd
A	−17.2	−17.1	−17.3	−17.2	−17.1	−17.2	0.1	9.1	9.1	9.2	9.1	9.2	9.1	0.0
B	−17.3	−17.4	−17.3	−17.4	−17.3	−17.3	0.1	8.5	8.6	8.2	8.7	8.6	8.5	0.2
C	−17.2	−17.0	−16.9	−17.0	−16.8	−17.0	0.1	9.2	8.7	8.9	8.7	8.9	8.9	0.2
D1	−17.2	−17.0	−17.1	−17.1	−17.2	−17.1	0.1	8.7	8.9	8.6	8.8	9.0	8.8	0.1
D2	−17.2	−17.1	−17.2	−17.2	−17.4	−17.2	0.1	8.6	8.6	8.6	8.8	9.1	8.7	0.2
F	−17.0	−17.0	−17.1	−16.9	−17.0	−17.0	0.1	9.1	9.1	9.3	9.3	8.8	9.1	0.2
G	−17.1	−16.6	−17.0	−17.2	−16.9	−16.9	0.2	9.0	9.0	9.0	9.2	9.0	9.1	0.1
H	−17.4	−16.8	−16.8	−17.1	−17.1	−17.0	0.2	9.5	9.6	9.7	9.9	9.4	9.6	0.2
I	−17.0	−17.0	−17.2	−17.0	−17.0	−17.0	0.1	9.3	9.4	9.3	9.3	9.2	9.3	0.1
J	−17.2	−17.4	−17.4	−17.3	−17.2	−17.3	0.1	8.9	8.9	8.8	9.0	8.8	8.9	0.1
K	−16.9	−17.1	−17.0	−17.1		−17.0	0.1	8.9	9.0	8.8	8.7		8.9	0.1
L	−17.0	−17.2	−17.7	−17.2	−17.1	−17.2	0.2	8.3	8.0	8.2	8.1	8.2	8.2	0.1
M	−16.9	−16.8	−16.9	−17.0	−17.1	−16.9	0.1	8.9	9.1	8.9	9.2	9.3	9.1	0.2
N	−17.0	−17.0	−17.0	−17.0	−17.1	−17.0	0.1	8.7	8.6	8.5	8.4	8.9	8.6	0.2
O	−17.2	−17.0				−17.1	0.1	9.0	9.4				9.2	0.2
P	−17.2	−17.2	−17.4	−17.3	−17.5	−17.3	0.1	9.2	9.3	9.4	9.4	9.3	9.3	0.1
Q	−16.2	−15.9	−16.2	−16.1	−16.1	−16.1	0.1	8.9	9.5	9.1	9.5	9.4	9.3	0.2
R	−17.1	−17.2	−17.0	−17.1	−17.1	−17.1	0.1	9.2	9.0	9.1	9.1	8.8	9.0	0.1
S	−17.1	−17.0	−17.1	−17.1	−17.0	−17.1	0.0	9.2	9.0	9.1	9.1	8.8	9.0	0.1
U	−16.9	−17.0	−17.0	−17.0	−17.0	−17.0	0.0	9.0	8.9	9.0	8.8	9.1	9.0	0.1
					Mean	−17.0						Mean	9.0	
					sd	0.3						sd	0.3	
					Median	−17.1						Median	9.0	
					Max	−15.9						Max	9.9	
					Min	−17.7						Min	8.0	

Analysis of inter-laboratory variation indicates significant differences among laboratories for the two isotope systems of interest. For δ^13^C_col_, the average pairwise inter-laboratory difference was 0.2‰ (Table 3, above diagonal), and the values obtained by the various laboratories were found to be significantly different (ANOVA, F_19,76_ = 19.3, p<0.01). Significant differences remained even after outliers were removed (ANOVA, F_18,71_ = 5.4, p<0.01). The average pairwise inter-laboratory difference for δ^15^N_col_ values was 0.4‰ (Table 3, below diagonal), with highly significant differences among laboratories (ANOVA, F_19,76_ = 19.3, p<0.01). Again, significant differences remained even after outliers were removed (ANOVA, F_19,69_ = 10.7, p<0.01).

**Table 3:** Difference matrix showing mean isotopic differences between laboratories for bone collagen results, δ^13^C_col_ above diagonal, δ^15^N_col_ beneath diagonal.

Lab			A	B	C	D1	D2	F	G	H	I	J	K	L	M	N	O	P	Q	R	S	U
	δ^13^C_col-VPDB_ (‰)		−17.2	−17.3	−17.0	−17.1	−17.2	−17.0	−16.9	−17.0	−17.0	−17.3	−17.0	−17.2	−16.9	−17.0	−17.1	−17.3	−16.1	−17.1	−17.1	−17.0
		δ^15^N_col-AIR_ (‰)	9.1	8.5	8.9	8.8	8.7	9.1	9.1	9.6	9.3	8.9	8.9	8.2	9.1	8.6	9.2	9.3	9.3	9.0	9.0	9.0
**A**	**−17.2**	**9.1**	-	0.1	0.2	0.1	0.0	0.2	0.2	0.2	0.2	0.1	0.2	0.0	0.3	0.2	0.1	0.1	1.1	0.1	0.1	0.2
**B**	**−17.3**	**8.5**	0.6	**-**	0.4	0.2	0.1	0.3	0.4	0.3	0.3	0.0	0.3	0.1	0.4	0.3	0.2	0.0	1.2	0.2	0.3	0.4
**C**	**−17.0**	**8.9**	0.2	0.4	-	0.1	0.2	0.0	0.0	0.1	0.1	0.3	0.0	0.2	0.0	0.0	0.1	0.3	0.9	0.1	0.1	0.0
**D1**	**−17.1**	**8.8**	0.3	0.3	0.1	-	0.1	0.1	0.2	0.1	0.1	0.2	0.1	0.1	0.2	0.1	0.0	0.2	1.0	0.0	0.1	0.1
**D2**	**−17.2**	**8.7**	0.4	0.2	0.1	0.1	-	0.2	0.3	0.2	0.2	0.1	0.2	0.0	0.3	0.2	0.1	0.1	1.1	0.1	0.1	0.2
**F**	**−17.0**	**9.1**	0.0	0.6	0.2	0.3	0.4	-	0.1	0.0	0.0	0.3	0.0	0.2	0.1	0.0	0.1	0.3	0.9	0.1	0.1	0.0
**G**	**−16.9**	**9.1**	0.1	0.5	0.2	0.3	0.3	0.1	-	0.1	0.1	0.4	0.1	0.3	0.0	0.0	0.2	0.4	0.8	0.2	0.1	0.0
**H**	**−17.0**	**9.6**	0.5	1.1	0.7	0.8	0.9	0.5	0.6	-	0.0	0.3	0.0	0.2	0.1	0.0	0.1	0.3	0.9	0.1	0.0	0.1
**I**	**−17.0**	**9.3**	0.2	0.8	0.4	0.5	0.6	0.2	0.2	0.3	-	0.3	0.0	0.2	0.1	0.0	0.1	0.3	0.9	0.1	0.0	0.1
**J**	**−17.3**	**8.9**	0.2	0.4	0.0	0.1	0.1	0.2	0.2	0.7	0.4	-	0.3	0.1	0.4	0.3	0.2	0.0	1.2	0.2	0.2	0.3
**K**	**−17.0**	**8.9**	0.3	0.3	0.0	0.0	0.1	0.3	0.2	0.8	0.5	0.0	-	0.2	0.1	0.0	0.1	0.3	0.9	0.1	0.0	0.0
**L**	**−17.2**	**8.2**	0.9	0.4	0.7	0.6	0.6	1.0	0.9	1.5	1.1	0.7	0.7	-	0.3	0.2	0.1	0.1	1.1	0.1	0.2	0.2
**M**	**−16.9**	**9.1**	0.0	0.6	0.2	0.3	0.3	0.0	0.0	0.5	0.2	0.2	0.2	0.9	-	0.1	0.2	0.4	0.8	0.2	0.1	0.0
**N**	**−17.0**	**8.6**	0.5	0.1	0.3	0.2	0.1	0.5	0.5	1.0	0.7	0.3	0.3	0.4	0.5	-	0.1	0.3	0.9	0.1	0.1	0.0
**O**	**−17.1**	**9.2**	0.1	0.7	0.3	0.4	0.5	0.1	0.1	0.4	0.1	0.3	0.4	1.0	0.1	0.6	-	0.2	1.0	0.0	0.0	0.1
**P**	**−17.3**	**9.3**	0.2	0.8	0.4	0.5	0.6	0.2	0.2	0.3	0.0	0.4	0.5	1.1	0.2	0.7	0.1	-	1.2	0.2	0.2	0.3
**Q**	**−16.1**	**9.3**	0.2	0.8	0.4	0.5	0.5	0.2	0.2	0.3	0.0	0.4	0.4	1.1	0.2	0.7	0.1	0.0	-	1.0	1.0	0.9
**R**	**−17.1**	**9.0**	0.1	0.5	0.2	0.2	0.3	0.1	0.0	0.6	0.3	0.2	0.2	0.9	0.0	0.4	0.2	0.3	0.2	-	0.0	0.1
**S**	**−17.1**	**9.0**	0.1	0.5	0.2	0.2	0.3	0.1	0.0	0.6	0.3	0.2	0.2	0.9	0.0	0.4	0.2	0.3	0.2	0.0	-	0.1
**U**	**−17.0**	**9.0**	0.2	0.4	0.1	0.2	0.2	0.2	0.1	0.7	0.3	0.1	0.1	0.8	0.1	0.4	0.2	0.3	0.3	0.1	0.1	-

It is worthwhile noting that neither the choice of demineralizing agent (HCl versus EDTA) nor the decision of whether/how to remove humic acids (NaOH, KOH, or no treatment) engendered significant differences in the resulting isotopic signatures. The offset seen between samples demineralized using HCl versus EDTA was only 0.2‰ for δ^13^C_col_ (t = 1.3, df = 94, p = 0.2) and 0.2‰ δ^15^N_col_ (t = 1.5, df = 94, p = 0.2). No significant differences in δ^13^C_col_ or δ^15^N_col_ were observed as a result of humic acid removal reagents; no treatment, NaOH, and KOH produced indistinguishable δ^13^C_col_ (ANOVA, F_2,93_ = 1.8, p = 0.2) and δ^15^N_col_ (ANOVA, F_2,93_ = 0.6, p = 0.5) values. It is possible that the lack of appreciable differences in isotope values between laboratories that did and did not remove humic acids could be a consequence of the sample's low initial humic content.

### Collagen reanalysis

**Figure 5:**
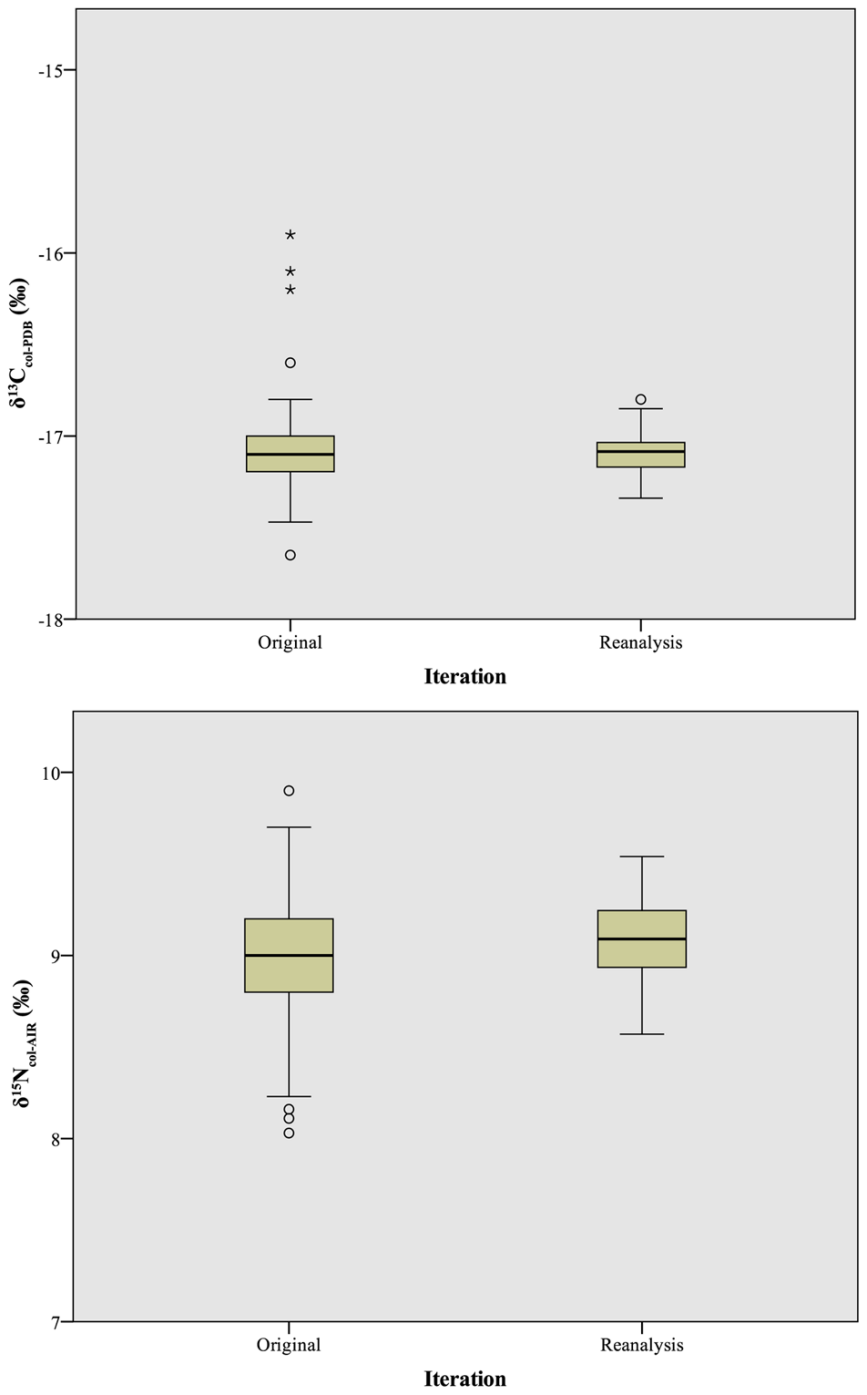
Boxplot comparison of δ^13^C_col_ (top) and δ^15^N_col_ (bottom) values between original analysis and reanalysis on a single instrument. Box lines represent first quartile, second quartile (median), and third quartile; whiskers at 95% confidence intervals; dots represent weak outliers (more than 2 standard deviations from mean); asterisks represent strong outliers (more than 3 standard deviations from mean).

Reanalysis of a subset of the collagen samples on the same instrument shifted both collagen isotope systems by approximately 0.1‰, with δ^13^C_col_ decreasing from -17.0±0.3‰ to −17.1±0.1‰ (t = 1.4, df = 145, p = 0.3; Table 4, Figure 5, top), and δ^15^N_col_ values increasing significantly (albeit not meaningfully) from 9.0±0.4‰ to 9.1±0.2‰ (t = 2.5, df = 145, p<0.01; Table 4, Figure 5, bottom). In both instances, reanalysis on the same instrument significantly reduced the variance for the measured samples. Standard deviation was significantly reduced for carbon (from 0.3‰ to 0.1‰; W = 8.4, df = 145, p<0.01) and nitrogen (0.4‰ to 0.2‰; W = 8.0, df = 145, p<0.01).

**Table 4:** Results of secondary reanalysis of bone collagen.

Lab	δ^13^C_col-VPDB_ (‰)					δ^15^N_col-AIR_ (‰)				
	R1	R2	R3	Mean	sd	R1	R2	R3	Mean	sd
A	−16.9	−17.0	−17.1	−17.0	0.1	9.0	9.2	9.3	9.2	0.2
B	−17.1	−17.3	−17.2	−17.2	0.1	9.2	9.0	8.9	9.0	0.2
D1	−17.1	−17.1	−17.1	−17.1	0.0	8.9	8.9	8.6	8.8	0.2
D2	−17.3	−17.1	−17.1	−17.2	0.1	9.0	8.8	8.9	8.9	0.1
F	−17.0	−17.2	−17.2	−17.1	0.1	8.6	8.9	9.0	8.8	0.2
G	−17.2	−17.2		−17.2	0.0	9.4	9.3		9.4	0.1
I	−17.2	−17.1	−17.0	−17.1	0.1	9.1	9.2	9.0	9.1	0.1
J	−17.1	−17.1	−17.0	−17.1	0.1	9.2	9.2	9.2	9.2	0.0
K	−17.0	−17.0	−17.1	−17.0	0.1	9.1	9.1	9.4	9.2	0.2
L	−16.9	−16.8	−17.3	−17.0	0.3	9.4	9.5	9.3	9.4	0.1
M	−17.2	−17.0	−17.2	−17.1	0.1	9.6	9.0	9.3	9.3	0.3
N	−17.0	−17.0	−17.0	−17.0	0.0	9.3	8.9	8.7	9.0	0.3
O	−17.1	−17.2		−17.2	0.1	9.2	8.7		9.0	0.4
P	−17.3	.17.2	−17.0	−17.2	0.2	8.9	9.0	8.9	8.9	0.1
Q	−17.3	−17.1	−16.9	−17.1	0.2	9.2	9.4	9.1	9.2	0.2
R	−17.1	−17.1	−16.9	−17.0	0.1	9.1	9.1	9.0	9.1	0.1
S	−17.0	−17.0	−17.0	−17.0	0.0	9.3	9.0	9.0	9.1	0.2
U	−17.1	−17.2	−17.1	−17.1	0.1	9.3	9.3	9.1	9.2	0.1
			Mean	−17.1				Mean	9.1	
			sd	0.1				sd	0.2	
			Median	−17.1				Median	9.1	
			Max	−16.8				Max	9.6	
			Min	−17.3				Min	8.6	

Reanalysis of the collagen samples affected the number and distribution of outliers. For δ^13^C_col_ (Figure 5, top), three samples, one each from Laboratories A, B, and L, possessed z-scores between 2.1 (p = 0.02) and 2.5 (p<0.01). For δ^15^N_col_ (Figures 5, bottom), a different set of three samples, one each from Laboratories D1, F, and M, had outlier z-scores between 2.0 (p = 0.02) and 2.3 (p = 0.01). Mean isotope values for laboratories that initially had uniformly outlying values (Laboratory Q for δ^13^C_col_ and Laboratory L for δ^15^N_col_) were no longer aberrant after reanalysis. This strongly suggests that although collagen pretreatment methods are responsible for some of the observed isotopic differences among laboratories, differences in instrumentation or data calibration also drive a large amount of the observed variation in isotope values among laboratories: 69% (1.2‰ of the initially observed 1.8‰ range) for δ^13^C_col_ and 48% (0.9‰ of the initially observed 1.9‰ range) for δ^15^N_col_ [59], [60].

### Establishing Minimum Meaningful Differences for Collagen

The Minimum Meaningful Difference (MMD) value, which takes into account both the average inter-laboratory difference and the typically observed intra-laboratory variability (in the form of the standard deviation of each laboratory's replicate measurements), was determined to be a modest 0.6‰ for δ^13^C_col_ (Table 5). This means that a difference in isotope values obtained from two different analyses is likely to be *bona fide* if that difference exceeds the threshold value of 0.6‰. MMD for δ^15^N_col_ (Table 5) was slightly higher (0.9‰). The relatively small magnitude of these values, as will be discussed below, provides substantial reassurance about the relative comparability of collagen isotope results obtained from different laboratories.

**Table 5:** Mean Measure of Difference values for four isotopic systems of interest.

Isotopic system	Average pairwise difference (‰)	Average intra-laboratory standard deviation (‰)	MMD (‰)
δ^13^C_ap_	0.6	0.15	1.2
δ^18^O_ap_	2	0.28	3.1
δ^13^C_col_	0.2	0.1	0.6
δ^15^N_col_	0.4	0.13	0.9

### Hydroxyapatite

**Figure 6:**
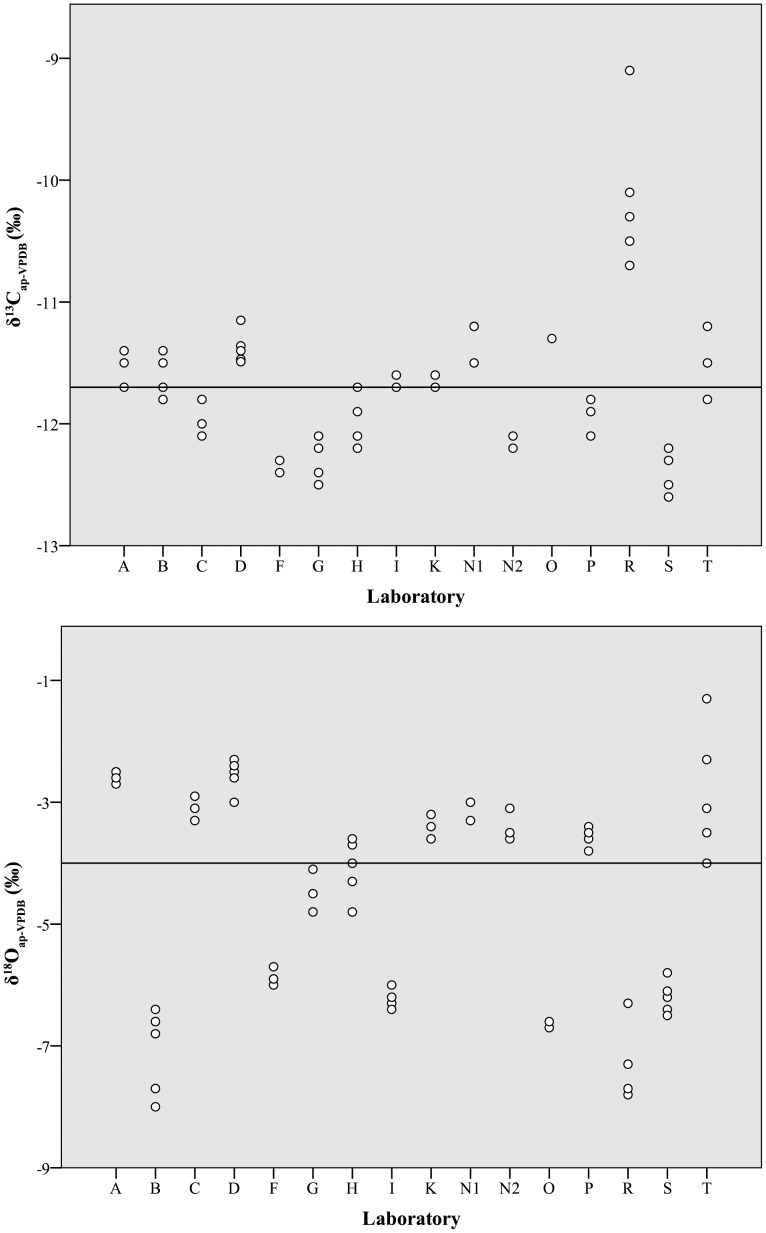
Distribution of initial δ^13^C_ap_ (top) and δ^18^O_ap_ (bottom) values by laboratory. Dots represent individual analyses and solid horizontal lines represent median values for all participating laboratories (−11.7‰ for δ^13^C_ap_ and −4.0‰ for δ^18^O_ap_).

**Figure 7:**
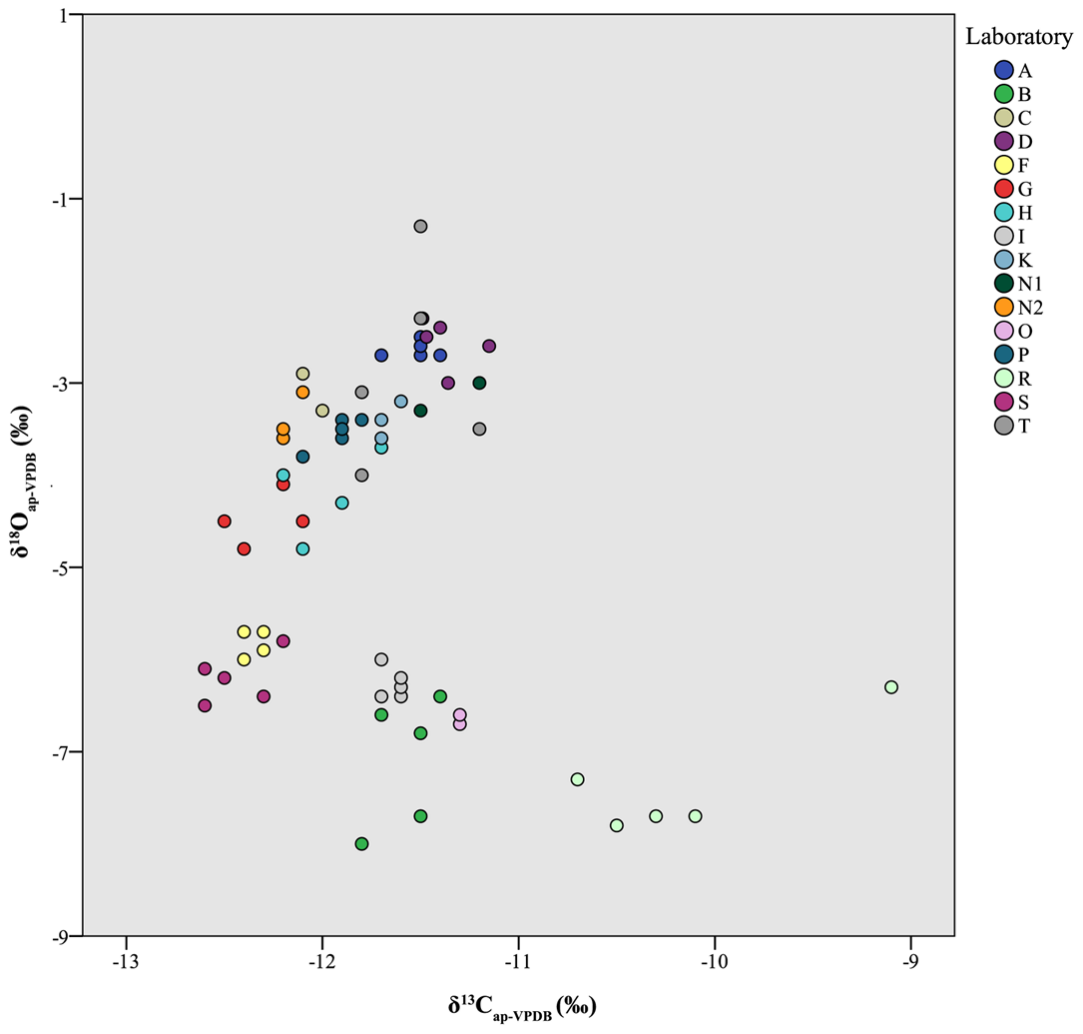
Scatterplot of individual δ^13^C_ap_ and δ^18^O_ap_ values presented for each laboratory. High variability of δ^18^O_ap_ values and outlying values of Laboratory R are both apparent.

Across all laboratories, δ^13^C_ap_ values averaged −11.7±0.6‰ and had an overall range of 3.5‰ (Table 6, Figure 6, top). Four measured values, all from the same laboratory (Laboratory R), were apparent outliers, with z-scores between 2.0 (p = 0.02) and 4.4 (p<0.01). Oxygen isotope values averaged −4.6±1.7‰, with an overall range of 6.7‰ (!) and no apparent outliers (Table 6, Figure 6, bottom). No significant Pearson correlation (r = −0.1, p = 0.2) was observed between δ^13^C_ap_ and δ^18^O_ap_ values (Figure 7).

**Table 6:** Results of initial analysis of bone hydroxyapatite.

Lab	δ^13^C_ap-VPDB_ (‰)							δ^18^O_ap-VPDB_ (‰)						
	R1	R2	R3	R4	R5	Mean	sd	R1	R2	R3	R4	R5	Mean	sd
A	−11.5	−11.4	−11.5	−11.7	−11.5	−11.5	0.1	−2.5	−2.7	−2.7	−2.7	−2.6	−2.6	0.1
B	−11.4	−11.7	−11.5	−11.8	−11.5	−11.6	0.1	−6.4	−6.6	−7.7	−8.0	−6.8	−7.1	0.6
C	−12.0	−12.1	−11.8			−12.0	0.1	−3.3	−2.9	−3.1			−3.1	0.2
D	−11.5	−11.2	−11.4	−11.5	−11.4	−11.4	0.1	−2.5	−2.6	−3.0	−2.3	−2.4	−2.5	0.2
F	−12.3	−12.4	−12.4	−12.4	−12.3	−12.4	0.0	−5.7	−5.7	−6.0	−6.0	−5.9	−5.9	0.1
G	−12.5	−12.1	−12.2	−12.4	−12.5	−12.3	0.2	−4.5	−4.5	−4.1	−4.8	−4.5	−4.5	0.2
H	−12.2	−11.7	−12.1	−11.9	−11.7	−11.9	0.2	−4.0	−3.7	−4.8	−4.3	−3.6	−4.1	0.4
I	−11.6	−11.6	−11.6	−11.7	−11.7	−11.7	0.1	−6.4	−6.3	−6.2	−6.4	−6.0	−6.2	0.2
K	−11.7	−11.6	−11.7	−11.7	−11.7	−11.7	0.1	−3.6	−3.2	−3.4	−3.4	−3.6	−3.4	0.2
N1	−11.5	−11.2				−11.4	0.2	−3.3	−3.0				−3.2	0.2
N2	−12.1	−12.2	−12.2			−12.2	0.0	−3.1	−3.6	−3.5			−3.4	0.2
O	−11.3	−11.3				−11.3	0.0	−6.7	−6.6				−6.7	0.0
P	−11.8	−11.9	−12.1	−11.9	−11.9	−11.9	0.1	−3.4	−3.4	−3.8	−3.6	−3.5	−3.5	0.2
R	−10.7	−10.3	−9.1	−10.5	−10.1	−10.1	0.6	−7.3	−7.7	−6.3	−7.8	−7.7	−7.4	0.6
S	−12.5	−12.3	−12.6	−12.6	−12.2	−12.4	0.2	−6.2	−6.4	−6.5	−6.1	−5.8	−6.2	0.3
T	−11.8	−11.8	−11.5	−11.2	−11.5	−11.6	0.2	−4.0	−3.1	−2.3	−3.5	−1.3	−2.8	0.9
					Mean	−11.7						Mean	−4.6	
					sd	0.6						sd	1.7	
					Median	−11.7						Median	−4.0	
					Max	−9.1						Max	−1.3	
					Min	−12.6						Min	−8.0	

**Figure 8:**
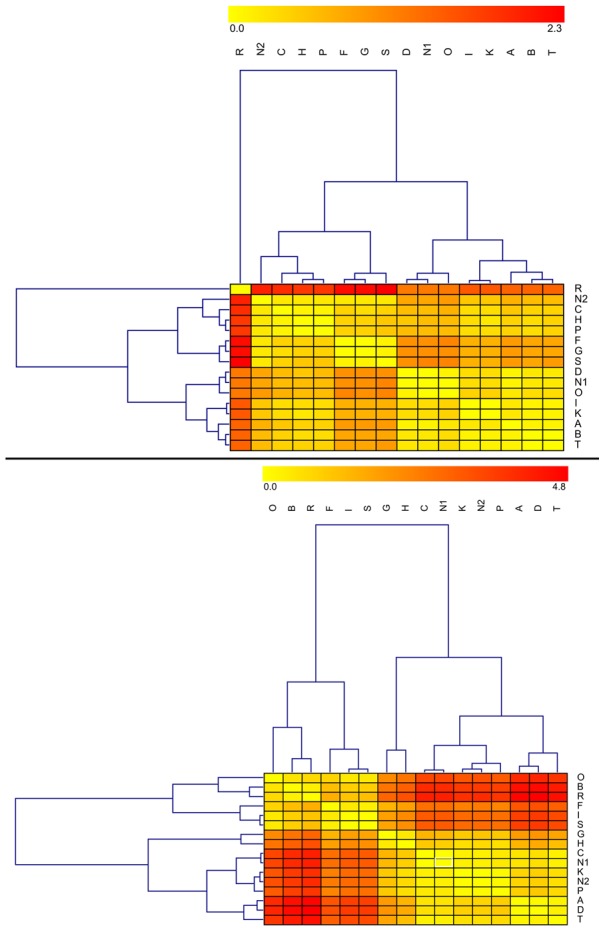
Heat maps of initial δ^13^C_ap_ (top) and δ^18^O_ap_ (bottom) values by laboratory. See Figure 3 legend for details.

Analysis of inter-laboratory variation indicates significant differences among laboratories for the two isotope systems of interest (note red cells in Figure 8). For δ^13^C_ap_, the average pairwise inter-laboratory difference was 0.6‰ (Table 7, above diagonal). Mean δ^13^C_ap_ values differed significantly among laboratories (ANOVA, F_15,54_ = 29.4, p<0.01), a finding that persisted following the removal of the four outlying values (ANOVA, F_14,50_ = 27.0, p<0.01). The average pairwise inter-laboratory difference for δ^18^O_ap_ values was 2.0‰ (Table 7, below diagonal), with highly significant differences in distribution among laboratories (ANOVA, F_15,54_ = 66.9, p<0.01).

**Table 7:** Difference matrix showing mean isotopic differences between laboratories for bone hydroxyapatite results; δ^13^C_ap_ above diagonal, δ^18^O_ap_ beneath diagonal.

Lab			A	B	C	D	F	G	H	I	K	N1	N2	O	P	R	S	T
	δ^13^C_ap-VPDB_ (‰)		−11.5	−11.6	−12.0	−11.4	−12.4	−12.3	−11.9	−11.7	−11.7	−11.4	−12.2	−11.3	−11.9	−10.1	−12.4	−11.6
		δ^18^O_ap-VPDB_ (‰)	−2.6	−7.1	−3.1	−2.5	−5.9	−4.5	−4.1	−6.2	−3.4	−3.2	−3.4	−6.7	−3.5	−7.4	−6.2	−2.8
**A**	−**11.5**	−**2.6**	-	0.1	0.5	0.1	0.9	0.9	0.4	0.2	0.2	0.1	0.7	0.2	0.4	1.4	0.9	0.1
**B**	−**11.6**	−**7.1**	4.5	-	0.4	0.2	0.8	0.8	0.3	0.1	0.1	0.2	0.6	0.3	0.3	1.4	0.8	0.0
**C**	−**12.0**	−**3.1**	0.5	4.0	-	0.6	0.4	0.3	0.1	0.3	0.3	0.6	0.2	0.7	0.1	1.9	0.4	0.4
**D**	−**11.4**	−**2.5**	0.1	4.6	0.6	-	1.0	1.0	0.5	0.3	0.3	0.0	0.8	0.1	0.6	1.2	1.0	0.2
**F**	−**12.4**	−**5.9**	3.2	1.2	2.7	3.3	-	0.0	0.4	0.7	0.7	1.0	0.2	1.1	0.4	2.2	0.1	0.8
**G**	−**12.3**	−**4.5**	1.9	2.6	1.4	2.0	1.4	-	0.4	0.7	0.7	1.0	0.2	1.0	0.4	2.2	0.1	0.8
**H**	−**11.9**	−**4.1**	1.4	3.0	1.0	1.6	1.8	0.4	-	0.3	0.2	0.6	0.2	0.6	0.0	1.8	0.5	0.4
**I**	−**11.7**	−**6.2**	3.6	0.9	3.1	3.7	0.4	1.8	2.2	-	0.0	0.3	0.5	0.4	0.3	1.5	0.7	0.1
**K**	−**11.7**	−**3.4**	0.8	3.7	0.3	0.9	2.4	1.0	0.6	2.8	-	0.3	0.5	0.4	0.3	1.5	0.7	0.1
**N1**	−**11.4**	−**3.2**	0.5	4.0	0.0	0.6	2.7	1.3	0.9	3.1	0.3	-	0.8	0.0	0.6	1.2	1.1	0.2
**N2**	−**12.2**	−**3.4**	0.8	3.7	0.3	0.9	2.5	1.1	0.7	2.8	0.0	0.3	-	0.9	0.2	2.0	0.2	0.6
**O**	−**11.3**	−**6.7**	4.0	0.5	3.5	4.1	0.8	2.2	2.6	0.4	3.2	3.5	3.3	-	0.6	1.2	1.1	0.3
**P**	−**11.9**	−**3.5**	0.9	3.6	0.4	1.0	2.3	1.0	0.6	2.7	0.1	0.4	0.1	3.1	-	1.8	0.5	0.4
**R**	−**10.1**	−**7.4**	4.7	0.3	4.2	4.8	1.5	2.9	3.3	1.1	3.9	4.2	4.0	0.7	3.8	-	2.3	1.4
**S**	−**12.4**	−**6.2**	3.6	0.9	3.1	3.7	0.3	1.7	2.1	0.0	2.8	3.1	2.8	0.4	2.7	1.2	-	0.9
**T**	−**11.6**	−**2.8**	0.2	4.3	0.3	0.3	3.0	1.7	1.2	3.4	0.6	0.3	0.6	3.8	0.7	4.5	3.4	-

**Figure 9:**
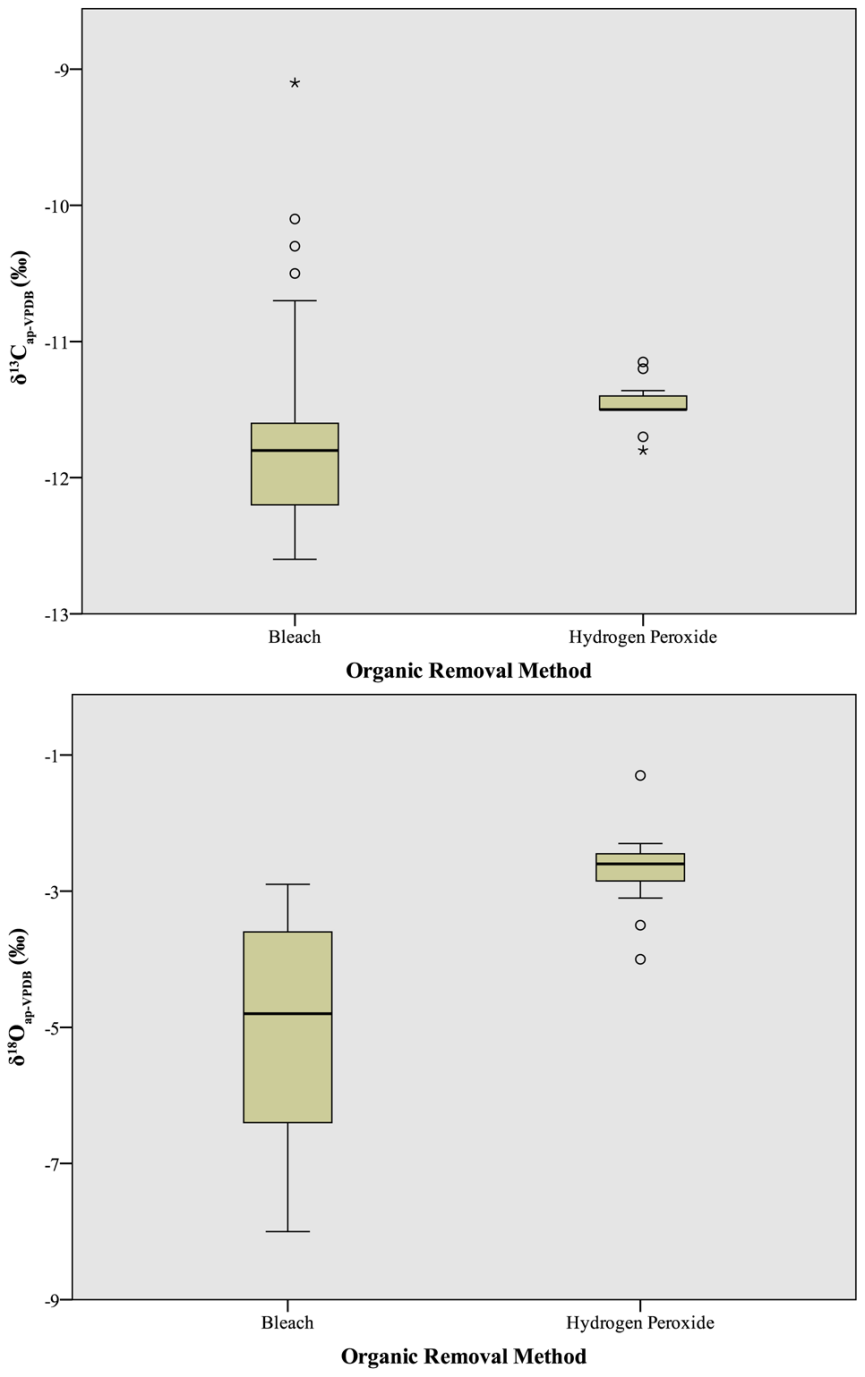
Boxplot comparison of δ^13^C_ap_ (top) and δ^18^O_ap_ (bottom) values obtained after oxidation with NaOCl versus H_2_O_2_. Box lines represent first quartile, second quartile (median), and third quartile; whiskers at 95% confidence intervals; dots represent weak outliers (more than 2 standard deviations from mean); asterisks represent strong outliers (more than 3 standard deviations from mean).

One presumed driver of inter-laboratory variation in isotope values is the chosen method of organic removal, namely bleach (sodium hypochlorite, NaOCl) versus hydrogen peroxide (H_2_O_2_). Indeed, the isotope values of samples processed using hydrogen peroxide produced significantly enriched δ^13^C_ap_ and δ^18^O_ap_ values compared to those samples processed with bleach, with values of −11.8±0.7‰ versus −11.5±0.2‰ for δ^13^C_ap_ (t = −2.9, df = 68, p<0.01; Figure 9, top), and −2.7±0.6‰ versus −5.1±1.6‰ for δ^18^O_ap_ (t = −9.2, df = 68, p<0.01; Figure 9, bottom). Indeed, it is this preparation difference that helps to explain why there are two clear clusters (high-level branchings) in the heat maps of Figure 8. As the difference in mean δ^18^O_ap_ values for samples processed by hydrogen peroxide versus bleach (2.4‰) is greater than the average pairwise difference in δ^18^O_ap_ values between any two participating laboratories (2.0‰), it would appear that the choice of reagent used for organic removal is a prime driver in inter-laboratory variation in δ^18^O_ap_ [41]. The same is not the case for δ^13^C_ap_, as the difference in means between oxidation methods (0.3‰) is less than the average pairwise inter-laboratory difference (0.6‰).

Differences in the labile carbonate removal technique (both concentration of acetic acid and the use of buffered versus un-buffered acetic) did not have a significant effect on δ^13^C_ap_ or δ^18^O_ap_ values. The offset seen between samples processed with 0.1–0.2 M versus 1.0 M acetic acid was only 0.03‰ for δ^13^C_ap_ (t = −0.19, df = 68, p = 0.9) and 0.15‰ δ^18^O_ap_ (t = −0.33, df = 68, p = 0.7). The differences between samples treated with buffered and un-buffered acetic acid were similarly small: 0.06‰ for δ^13^C_ap_ (t = 0.37, df = 68, p = 0.7) and 0.2‰ for δ^18^O_ap_ (t = −0.49, df = 68, p = 0.6). This result is somewhat unexpected, as previous studies [22], [42] have reported that acid strength has an impact on δ^13^C_ap_ and δ^18^O_ap_ values.

### Hydroxyapatite reanalysis

**Figure 10:**
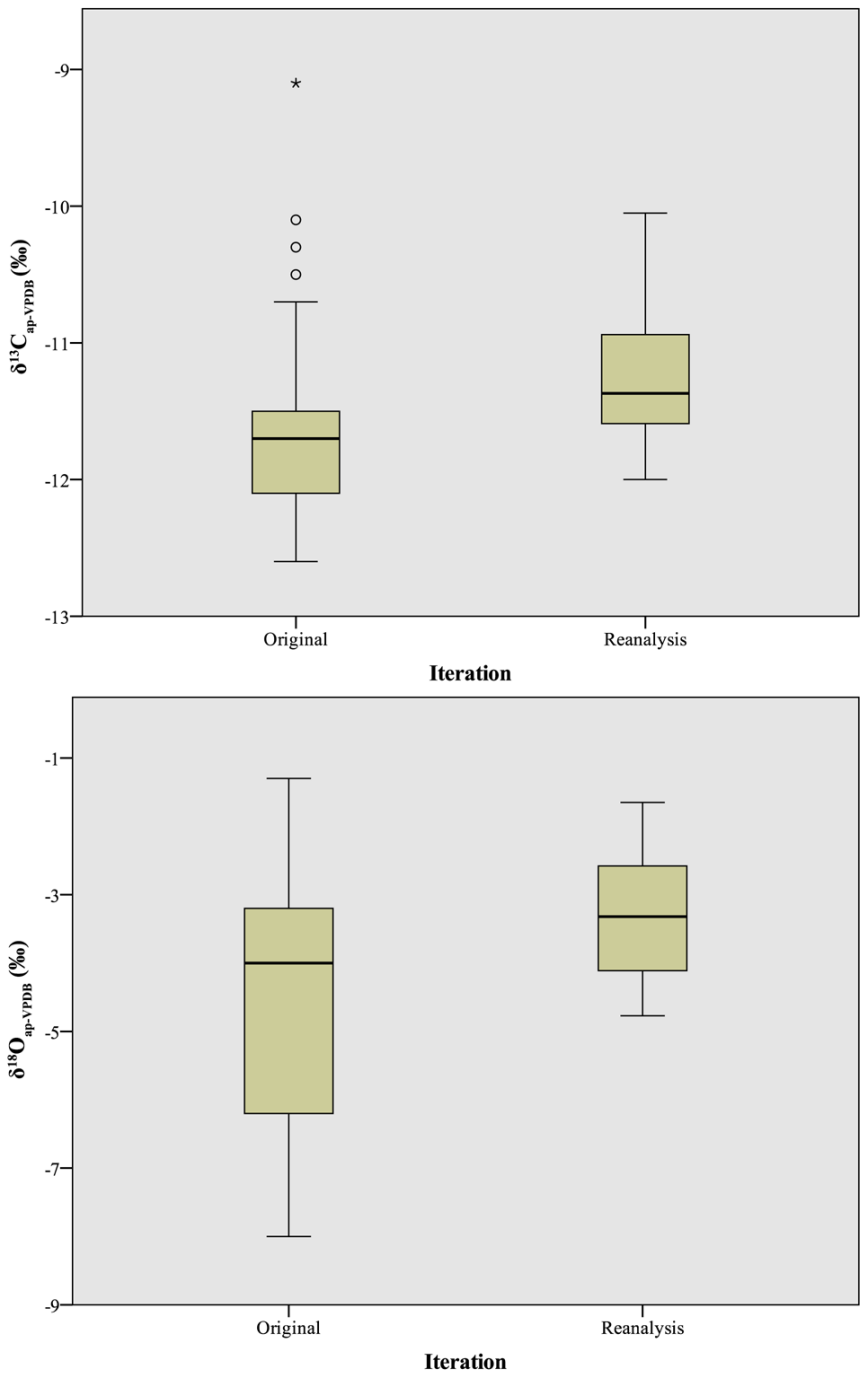
Boxplot comparison of δ^13^C_ap_ (top) and δ^18^O_ap_ (bottom) values between original analysis and reanalysis on a single instrument. Box lines represent first quartile, second quartile (median), and third quartile; whiskers at 95% confidence intervals; dots represent weak outliers (more than 2 standard deviations from mean); asterisks represent strong outliers (more than 3 standard deviations from mean).

Subsequent reanalysis of a subset of the hydroxyapatite samples on the same instrument revealed at least two interesting phenomena. First, reanalysis produced significantly enriched isotope results for carbon isotope values, which increased modestly from −11.7±0.6‰ to −11.2±0.5‰ (t = −4.0, df = 99, p<0.01; Table 8, Figure 10, top), as well as oxygen isotope values, which increased more dramatically from −4.6±1.7‰ to −3.4±0.9‰ (t = −4.6, df = 99, p<0.01; Table 8, Figure 10, bottom). Second, reanalysis on the same instrumentation significantly reduced the variance for δ^18^O_ap_, almost halving the standard deviation of the measured samples from 1.7‰ to 0.9‰ (W = 25.3, df = 99, p<0.01). While the variance for δ^13^C_ap_ was also reduced during reanalysis, the observed difference (0.6‰ versus 0.5‰) was modest by comparison, and not significant (W = 0.01, df = 92, p = 0.92).

**Table 8:** Results of secondary reanalysis of bone hydroxyapatite.

Lab	δ^13^C_ap-VPDB_ (‰)					δ^18^O_ap-VPDB_ (‰)				
	R1	R2	R3	Mean	sd	R1	R2	R3	Mean	sd
A	−11.4	−11.8	−11.4	−11.5	0.2	−2.3	−3.5	−2.3	−2.7	0.7
B	−10.9	−10.9	−11.3	−11.0	0.2	−4.3	−4.8	−4.1	−4.4	0.3
F	−11.6	−11.5	−11.6	−11.6	0.1	−3.0	−2.5	−3.0	−2.9	0.3
I	−11.6	−11.0	−11.4	−11.3	0.3	−4.7	−4.0	−4.4	−4.4	0.3
K	−11.7	−11.5	−11.6	−11.6	0.1	−2.7	−2.2	−2.3	−2.4	0.2
N2	−11.0	−11.3		−11.2	0.2	−3.2	−3.0		−3.1	0.1
O	−10.0	−10.3		−10.2	0.1	−3.7	−4.1		−3.9	0.3
P	−11.8	−12.0	−11.2	−11.7	0.4	−3.7	−3.7	−3.3	−3.6	0.2
R	−10.8	−11.2	−11.5	−11.2	0.4	−4.5	−4.7	−4.5	−4.5	0.1
S	−11.6	−10.7	−11.7	−11.4	0.5	−3.3	−2.7	−4.1	−3.4	0.7
T	−10.3	−10.4	−10.9	−10.6	0.3	−1.7	−1.8	−2.4	−2.0	0.4
			Mean	−11.2				Mean	−3.4	
			sd	0.5				sd	0.9	
			Median	−11.4				Median	−3.3	
			Max	−10.0				Max	−1.7	
			Min	−12.0				Min	−4.8	

Two other phenomena of note were evident after reanalysis. First, the samples from Laboratory R, which had outlying δ^13^C_ap_ values in the initial run, were not outliers in the reanalysis (z-scores between −0.5 [p = 0.3] and 0.9 [p = 0.18]). Indeed, there was only one outlier among the two isotope systems, a solitary samples from Laboratory O which had a δ^13^C_ap_ value with a z-score of 2.4 (p<0.01). This finding suggests that Laboratory R's aberrant isotope values in the first round of analysis were the result of analytical instrumentation, working standards, or data calibration rather than a preparation step [59], [60]. Indeed, these factors, rather than pretreatment *per se*, would seem to drive a large portion of the observed variation in isotope values for hydroxyapatite: 44% (1.5‰ of the initially observed 3.5‰ range) for δ^13^C_ap_ and 54% (3.6‰ of the initially observed 6.7‰ range) for δ^18^O_ap_. These results echo the recent findings of Carter and Fry [59] who demonstrated that differences in data calibration and correction can lead to substantial isotopic differences among laboratories.

Second, while reanalysis on the same instrument reduced the difference in δ^13^C_ap_ values between samples oxidized using bleach versus hydrogen peroxide (<0.3‰, t = −1.1, df = 29, p = 0.3), a significant difference in δ^18^O_ap_ values remained (1.3‰, t = −3.6, df = 29, p<0.01). Therefore, although instrumentation drove some of the isotopic variation among laboratories, differences in preparation (particularly oxidation/organic removal) were also responsible for observed differences in isotopic values obtained from different laboratories, a finding that is in agreement with previous studies [41], [43].

### Establishing Meaningful Minimum Differences for Hydroxyapatite

As noted above, the average inter-laboratory pairwise differences for δ^13^C_ap_ and δ^18^O_ap_ were 0.6‰ and 2.0‰ respectively. The Minimum Meaningful Difference (MMD) value for δ^13^C_ap_ was determined to be 1.2‰ (Table 5). This value suggests that a difference in isotopic signatures obtained from two different analyses is only likely to be *bona fide* when that difference exceeds the threshold value of 1.2‰. MMD for δ^18^O_ap_ (Table 5) was much larger (3.1‰). This latter value is of particular concern, as it is greater than the difference in *bona fide* δ^18^O values that might be expected to result from biological or environmental differences (e.g., residency, residency or paleoclimate), as is more fully elucidated below.

## Conclusions

The present study began with the goals of: 1) quantifying inter-laboratory variability in stable isotope analysis of bone collagen and hydroxyapatite, and 2) tracing the likely causes of this observed variability. For bone collagen, we found statistically significant inter-laboratory variation for both carbon and nitrogen isotope values among laboratories. However, the average pairwise difference between any two participating laboratories was only 0.2‰ for δ^13^C_col_ and 0.4‰ for δ^15^N_col_. These values are of such a small magnitude as to not be cause for great concern. As to causality, neither of the most obvious differences in pretreatment between participating laboratories (demineralizing reagent or humic acid removal) had a significant effect on the resulting isotope values.

Subsequent reanalysis of a subset of samples on the same instrument indicates that the prime driver of inter-laboratory variation in collagen stable isotope analysis is differences in analytical instrumentation and/or standardization rather than pretreatment (accounting for 48–69% of the observed initial inter-laboratory variation). Finally, the Minimum Meaningful Difference (MMD) value establishes a threshold by which results obtained from two laboratories might be evaluated. Differences exceeding 0.6‰ for δ^13^C_col_ and 0.9‰ for δ^15^N_col_ have a high likelihood of being of biological origin rather than an artifact of pretreatment or analysis. In sum, it would appear that the results of stable isotope analysis of bone collagen from one laboratory can be compared (if cautiously) with results obtained elsewhere. Overall, inter-laboratory variability in collagen isotopes would not appear to be of paramount concern.

For bone hydroxyapatite, the results of the present study are somewhat less reassuring. Inter-laboratory variability for both δ^13^C_ap_ and δ^18^O_ap_ was significant, and while the average pairwise difference between any two participating laboratories was only 0.6‰ for δ^13^C_ap_, for δ^18^O_ap_, that value rose to 2.0‰, a difference which could easily change interpretations of past residency or paleomobility. It is unlikely that anything more than a small portion of this variability is the result of differential diagenesis [43], as variability within laboratories (each of which received a randomized set of bone samples) was significantly less than inter-laboratory variability. Instead, as previously suggested [41], differences in oxidation treatment (NaOCl versus H_2_O_2_) appear to be a prime driver of δ^18^O_ap_ variability, but not for δ^13^C_ap_. However, differences in the method for removing labile carbonate (acid concentration and buffering agent) do not have a significant effect on either isotope system, counter previous suggestions [22], [42] to the contrary. Perhaps such difference were not observed because all laboratories that used strong acid used buffered acid or very short treatment times.

As with collagen, the subsequent reanalysis of a subset of samples on the same instrument indicates that differences in analytical instrumentation and/or standardization (rather than pretreatment per se) were a prime driver of inter-laboratory variation in hydroxyapatite stable isotope values (accounting for 44–54% of the observed initial inter-laboratory variation). Efforts to unify data correction among laboratories would likely decrease this variability [59], [60]. The Minimum Meaningful Difference (MMD) values suggest that results obtained from two laboratories have a high likelihood of being *bona fide* rather than an artifact of different pretreatment or analytical methods only if they exceed 1.2‰ for δ^13^C_ap_ and 3.1‰ for δ^18^O_ap_. The magnitude of these MDD values, particularly for δ^18^O_ap_, might call into question the attribution of biological significance oftentimes given to different δ^13^C_ap_ and δ^18^O_ap_ values obtained in different laboratories. In sum, it would appear that inter-laboratory variability could be a significant concern for hydroxyapatite. Analytical results from different laboratories might not be directly comparable, particularly in the case of δ^18^O_ap_.

Three final points merit consideration. First, it should be noted that while the present study addresses the wisdom of (over)claiming the significance of dissimilar results from different laboratories, even small differences (if replicable) obtained in one laboratory can still be considered reliable. Second, the bone hydroxyapatite results presented here may not be directly applicable to comparisons of enamel hydroxyapatite, a tissue thought to be far more resistant to diagenesis [43]. However, the results presented here may be used as a cautionary starting point for enamel comparisons. Third, and finally, the results presented here ought to be thought of in terms of providing a minimum estimate of potential variability that could be generated among laboratories. The extremely high quality of preservation of the selected ancient bone specimen might lead us to underestimate possible inter-laboratory variation in preparation methods. We would expect to see larger isotopic differences among laboratories if they prepare and analyze a poorly preserved sample with lower collagen yield, greater post-mortem humic contamination, or more non lattice-bound carbonates.

## Supporting Information

Figure S1 **Sample hydroxyapatite extraction protocol form.**
(EPS)

Figure S2 **Sample hydroxyapatite result form.**
(EPS)

Figure S3 **Sample collagen extraction protocol form.**
(EPS)

Figure S4 **Sample collagen result form.**
(EPS)

Table S1 **Summary of the collagen pretreatment methods for each participating laboratory.**
(XLSX)

Table S2 **Summary of the hydroxyapatite pretreatment methods for each participating laboratory.**
(XLSX)
